# Increased Expression of SERPINB10 Associated with Postoperative Recurrence in Chronic Rhinosinusitis with Nasal Polyps

**DOI:** 10.1155/2022/7164318

**Published:** 2022-11-08

**Authors:** Zhenghao Deng, Zhi Li, Yongchuan She, Bin Xie

**Affiliations:** ^1^Department of Pathology, Xiangya Hospital of Central South University, Changsha, Hunan, China; ^2^National Clinical Research Center for Geriatric Disorders, Xiangya Hospital of Central South University, Changsha, Hunan, China; ^3^Department of Pathology, School of Basic Medicine, Central South University, Changsha, Hunan, China; ^4^Department of Pathology, Third Xiangya Hospital of Central South University, Changsha, Hunan, China; ^5^Department of Otolaryngology Head and Neck Surgery, Changsha Hospital of Traditional Chinese Medicine, Changsha, Hunan, China

## Abstract

**Background:**

Chronic rhinosinusitis with nasal polyps (CRSwNP) is a common upper airway inflammatory disorder with a high rate of postoperative recurrence. SERPINB10 is a proinflammatory cytokine expressed on epithelial cells, but its role in CRSwNP has not been described. This study is aimed at exploring the SERPINB10 expression in CRSwNP and its relationship with postoperative recidivation.

**Methods:**

We recruited 140 individuals, consisting of 60 patients with CRSwNP, 40 patients with chronic rhinosinusitis without nasal polyps (CRSsNP), and 40 healthy controls (HCs). Tissue specimens were collected during the surgery, and SERPINB10 expression was determined by reverse transcription-polymerase chain reaction, western blotting, and immunofluorescence. We determined the tissue SERPINB10 expression levels in CRSwNP and examined its clinical value in predicting postoperative recurrence.

**Results:**

We determined that tissue SERPINB10 mRNA and protein levels were increased in the CRSwNP group, especially in the recurrent CRSwNP group, compared with the CRSsNP and HC groups (*p* < 0.05), and SERPINB10 mRNA levels were correlated with peripheral and tissue eosinophil counts and percentages (*p* < 0.05). Binary logistic regression analysis and receiver operating characteristic (ROC) curves suggested that the expressions of tissue SERPINB10 mRNA were significantly linked to postoperative recurrence in CRSwNP patients (AUC = 0.741, *p* < 0.001).

**Conclusion:**

Elevated local SERPINB10 levels in patients with CRSwNP were related to tissue eosinophilic inflammation and disease recurrence. These data suggested that SERPINB10 might contribute to the eosinophilic inflammation in CRSwNP and appeared to be a potential biomarker for the prediction of relapse after surgery.

## 1. Introduction

Chronic rhinosinusitis with nasal polyp (CRSwNP) is a frequent chronic inflammatory disease in the upper airway with a steadily increasing prevalence [1, 2]. The representative symptoms of CRSwNP consist of nasal obstruction, olfactory dysfunction, rhinorrhea, and facial pain [3, 4]. Previous studies indicated that the dominant tissue pathological feature of CRSwNP was T helper 2- (Th2-) type inflammation and eosinophil accumulation, which might contribute to a high tendency of postoperative recurrence [5–7]. Although functional endoscopic sinus surgery (FESS) is proven to significantly improve clinical symptoms and nasal inflammation for CRSwNP, many patients still suffer a recurrence during the follow-up [8, 9]. Previous studies demonstrated that relapse rate of CRSwNP was fluctuant, varying from 4% to 60%, with a median of 20% over a maximum of 2 years [10, 11]. As a clinical problem with a remarkably high recurrence rate, discovering novel biomarkers to predict postoperative recurrence of CRSwNP is extremely important. Taken together, identifying objective methods or biomarkers to predict postoperative recurrence is desperately needed and clinically meaningful.

Serine protease inhibitors (SERPINS) are a superfamily of homologous proteins, and it has been shown to present essential roles in inflammation, immune response, and regulation of apoptosis [12, 13]. Previous studies found that human SERPINS could be categorized into 9 groups (A-I), named “clades,” based on their sequence similarity, and some members were demonstrated to be overexpressed in allergic diseases via regulating Th1/Th2 responses [14–16]. SERPINB10, as another member of clade B, has been proven to be increased in bronchial epithelial cells of asthmatic patients and associated with airway Th2 and eosinophilic inflammation [17]. Moreover, Mo et al. [18] revealed that epithelial SERPINB10 might serve as a new biomarker for reflecting the degree of airway eosinophilic inflammation in asthma. However, whether SERPINB10 is involved in the pathological process of CRSwNP remains unclear. Therefore, this study is aimed at investigating the expression levels of SERPINB10 in CRSwNP patients and elevating its predictive ability for recurrence.

## 2. Methods

### 2.1. Patients and Sample Collections

We recruited 40 CRSsNP patients and 60 CRSwNP patients (36 primary CRSwNP (pCRSwNP) and 24 recurrent CRSwNP(rCRSwNP)) who underwent FESS between March 2021 and September 2021. The diagnosis of CRSwNP was performed referring to the clinical definition of chronic rhinosinusitis in adults, EPOS 2012 [19]. Exclusion criteria were listed as follows: (1) with fungal sinusitis, allergic fungal rhinitis, or malignancy; (2) with autoimmune disease and other allergic or eosinophilic disease; (3) received immunotherapy, antibiotics, nasal or systemic corticosteroids, or antiallergic medications four weeks before recruitment; and (4) patients < 18 years of age or >70 years of age. In addition, 40 patients who underwent septoplasty without other nasal disorders were enrolled as healthy controls (HCs), and their mucosa of the middle turbinate was harvested.

### 2.2. Real-Time PCR

Real-time PCR was conducted as described previously [20]. Briefly, total RNA was extracted from collected tissue samples using with Ncmzol reagent (New Cell & Molecular Biotech, Suzhou, China). Total RNA (1 *μ*g) was reverse transcribed into cDNA. Real-time PCR amplification and detection were performed according to the manufacturer's protocol. The expression of the target gene was normalized to the expression of 3-phosphate dehydrogenase (GAPDH). The primer sequences used were as follows: GAPDH: forward primer (5′-3′) TCGCTCAGACACCATGGGGAAGGT and reverse primer (5′-3′) TGAGCTCTCCTTGCGGGGAACA and SERPINB10: forward primer (5′-3′) GGACGGTTGAGCCAAAACCCTTCA and reverse primer (5′-3′) CCCATGAAATGGTGGCCAGGAGA.

### 2.3. Western Blotting Analysis

The western blotting is operated as previously described [20, 21]. In brief, the total protein was extracted from collected tissue specimens, and protein concentrations were measured. The extracted proteins were separated by SDS-PAGE gel electrophoresis (Beyotime Biotech, Shanghai, China) and transferred on nitrocellulose membranes. The transfer membranes were blocked with 5% nonfat milk for 1 h at room temperature. Primary antibodies against SERPINB10 (1 : 1000) and anti-GAPDH (1 : 3000) (Affinity Biosciences, Changzhou, China) were used to incubate the membranes overnight at 4°C. Then, membranes were incubated with peroxidase-conjugated secondary anti-rabbit IgG (Affinity Biosciences, Changzhou, China) for 2 h at room temperature followed by ECL western blot detection reagents (New Cell & Molecular Biotech, Suzhou, China). Densitometry was conducted by applying ImageJ (National Institutes of Health, USA), and protein levels of SERPINB10 were indexed to GAPDH.

### 2.4. Immunofluorescence Staining of SERPINB10

To further determine the location and level of SERPINB10 expression in tissues, we used immunofluorescence analysis, which was performed as described previously [22]. Primary antibodies included anti-SERPINB10. After 24 h of incubation with primary antibodies at 4°C, the sections were incubated with secondary antibodies (Affinity Biosciences, Changzhou, China) for 1 hour at room temperature. Then, sections were incubated with 4,6-diamidino-2-phenylindole (DAPI, Beyotime Biotechnology, Shanghai, China) for 2 minutes for visualization of cell nuclei. The proportion of SERPINB10 positive cells was calculated and analysed by two independent observers.

### 2.5. Statistical Analysis

All numerical variables were shown as mean ± standard deviation (SD). For normally distributed variables, Student's *t*-test and ANOVA were applied to difference comparison between two groups and among three groups, respectively; otherwise, the Mann-Whitney *U* test and Wilcoxon rank sum test were conducted. Correlations between tissue SERPINB10 mRNA and clinical variables were examined by Spearman's rank correlation. Binary regression analysis was performed to confirm the relationship between tissue SERPINB10 mRNA and postoperative recurrence, and receiver operating characteristic (ROC) curves were conducted to evaluate its predictive capacity. SPSS statistical software version 25.0 (IBM, Chicago, IL, USA) was applied for statistical analysis, and *p* < 0.05 was considered statistically significant.

## 3. Results

### 3.1. Patients' Demographics

The demographics and clinical variables of all subjects are shown in Table 1. The patients in the CRSwNP group exhibited a higher percentage of accompanying allergic rhinitis and asthma and blood eosinophil count and percentage than the CRSsNP and HC groups (all *p* < 0.05). Moreover, the VAS score and TNSS score were greater in the CRSwNP group than the CRSsNP group (all *p* < 0.05).  Table 2 indicates that recurrent CRSwNP patients presented a higher rate of allergic rhinitis, peripheral eosinophil percentage, tissue eosinophil count and percentage, and Lund-Mackay score than primary subjects (all *p* < 0.05).

### 3.2. The mRNA and Protein Levels of Tissue SERPINB10 in CRSwNP and Their Associations with Eosinophil Inflammation

**Figure 1:**
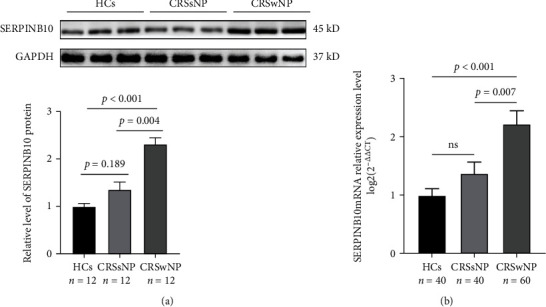
The expression levels of SERPINB10 in different groups. Tissue SERPINB10 protein (a) and mRNA levels (b) were significantly raised in the CRSwNP patients than the HCs and CRSsNP patients. HC: health control; CRSwNP: chronic rhinosinusitis with nasal polyp; CRSsNP: chronic rhinosinusitis without nasal polyp.

**Figure 2:**
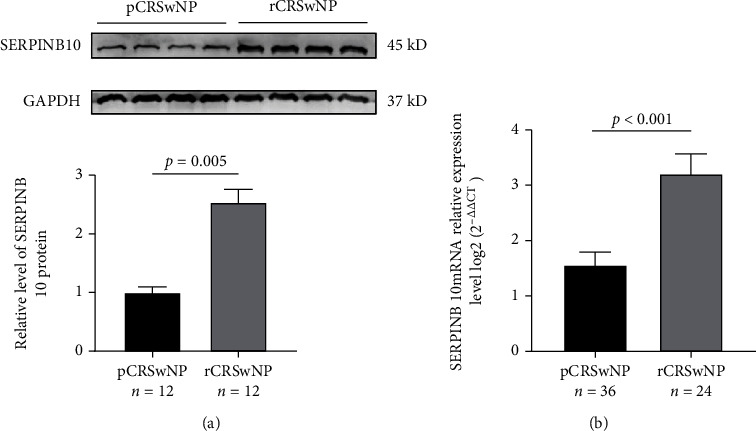
The expression levels of SERPINB10 in the recurrent and primary groups. Tissue SERPINB10 protein (a) and mRNA levels (b) were clearly enhanced in the rCRSwNP patients than pCRSwNP patients. pCRSwNP: primary chronic rhinosinusitis with nasal polyp; rCRSwNP: recurrent chronic rhinosinusitis with nasal polyp.

**Figure 3:**
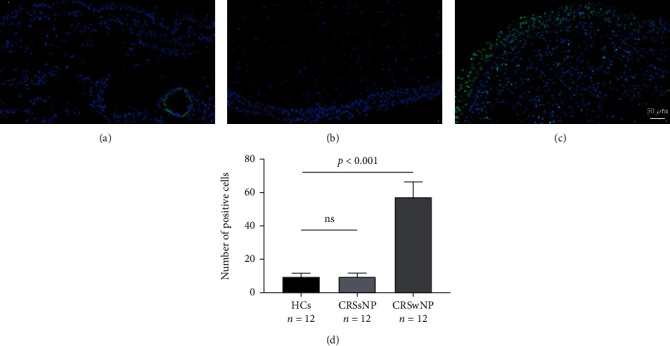
The expressions of SERPINB10+ cell in the nasal tissues from HCs (a), CRSsNP patients (b), and CRSwNP patients (c) were stained by immunofluorescence. Compared with the HCs and CRSsNP group, tissue SERPINB10+ cell numbers were notably higher in the CRSwNP group (d). HC: health control; CRSwNP: chronic rhinosinusitis with nasal polyp; CRSsNP: chronic rhinosinusitis without nasal polyp.

**Figure 4:**
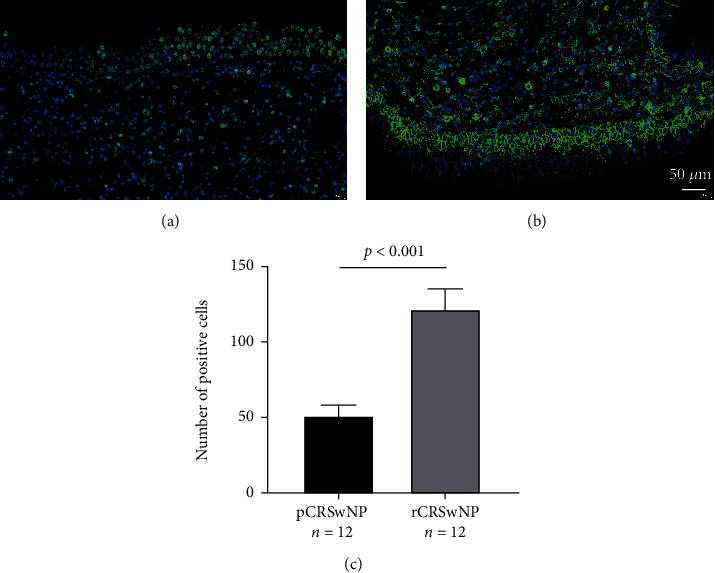
The expression of SERPINB10+ cell in the pCRSwNP group (a) and rCRSwNP group (b). Tissue SERPINB10+ cell numbers in the pCRSwNP group were remarkably increased than pCRSwNP group (c). pCRSwNP: primary chronic rhinosinusitis with nasal polyp; rCRSwNP: recurrent chronic rhinosinusitis with nasal polyp.

**Figure 5:**
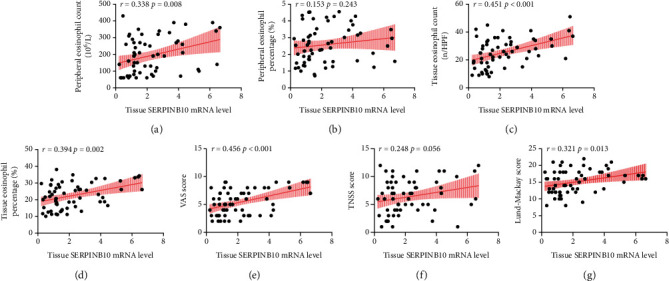
Correlations between the tissue SERPINB10 mRNA levels and clinical parameters in CRSwNP patients. Tissue SERPINB10 mRNA levels correlated with peripheral eosinophil count (a), tissue eosinophil count (c) and percentage (d), VAS score (e), and Lund-Mackay score (g). Tissue SERPINB10 mRNA levels have no correlation with peripheral eosinophil percentage (b) and TNSS score (f). CRSwNP: chronic rhinosinusitis with nasal polyp; VAS: visual analogue scale; TNSS: total nasal symptom score.

As displayed in Figure 1, both tissue SERPINB10 mRNA and protein were enhanced in the CRSwNP group than the HC and CRSsNP groups (*p* < 0.05). Moreover, compared with the pCRSwNP group, the protein and mRNA levels of tissue SERPINB10 were significantly higher in the rCRSwNP (Figure 2). To further explore distribution of SERPINB10 in the nasal mucosa, IF was performed. The results demonstrated that the staining of SERPINB10 was mainly situated in the epithelial and submucosal regions of the nasal cavity. More significant SERPINB10 immunoreactivity was detected in tissue from CRSwNP than CRSsNP patients and HCs (Figure 3), and the immunoreactivity of SERPINB10 was distinctly augmented in relapsed patients compared to primary patients (Figure 4). In addition, we found positive correlation between tissue SERPINB10 mRNA levels and blood eosinophil count (*r* = 0.338, *p* = 0.008), tissue eosinophil count (*r* = 0.451, *p* < 0.001) and percentage (*r* = 0.394, *p* = 0.002), and Lund-Mackay score (*r* = 0.321, *p* = 0.013) (Figure 5). The detailed data is shown in Table 3.

### 3.3. Evaluating the Predictive Ability of Tissue SERPINB10 for Postoperative Recurrence

**Figure 6:**
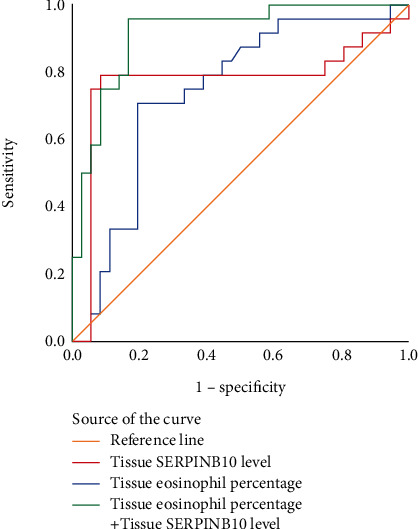
ROC curves of the tissue SERPINB10 mRNA levels (red line), percentage of eosinophils in polyp tissue samples (blue line), and combination of tissue SERPINB10 mRNA levels and tissue eosinophil percentage (green line). The AUC value of tissue SERPINB10 (AUC = 0.773, *p* < 0.001) was significantly higher than tissue eosinophil percentage (AUC = 0.741, *p* = 0.002). ROC: receiver operating characteristic; AUC: the area under the curve.

The binary logistic regression analysis suggested that tissue eosinophil percentage (OR = 1.176, *p* = 0.016) and tissue SERPINB10 mRNA levels (OR = 1.982, *p* = 0.014) were related to CRSwNP recurrence (Table 4). ROC curves in Figure 6 indicated that tissue SERPINB10 mRNA levels (AUC = 0.773, 95% CI 0.625-0.921, *p* < 0.001) exhibited stronger predictive capability for CRSwNP recidivation ability than tissue eosinophil percentage (AUC = 0.741, 95% CI 0.612-0.871, *p* = 0.002). Moreover, the combination of tissue SERPINB10 mRNA levels and tissue eosinophil percentage presented greater predictive efficacy (AUC = 0.917, 95% CI 0.845-0.989, *p* < 0.001). The detailed data is presented in Table 5.

## 4. Discussion

CRSwNP is a highly heterogeneous disorder with a high rate of postoperative recurrence [1, 23, 24]. Although great efforts have been made to explore the pathogenesis of CRSwNP, an early prediction system for of CRSwNP recurrence has still not been constructed. Therefore, exploring predictive biomarkers for prognosis and recurrence of CRSwNP is crucial, especially for those patients prone to recurrence. In the present study, we demonstrated that tissue SERPINB10 expression levels were clearly elevated in CRSwNP patients, especially in recurrent patients. Moreover, SERPINB10 expressions were strongly correlated with the degree of tissue eosinophilic inflammation and the risk of recurrence after surgery. ROC curve indicated that tissue SERPINB10 mRNA levels exhibited a strong predictive capability for CRSwNP recurrence. Given that, our findings hinted that SERPINB10 might be involved in the pathogenesis of CRSwNP and contribute to tissue eosinophilic inflammation. Thereby, tissue SERPINB10 appeared to be a novel indicator for predicting postoperative relapse in CRSwNP patients.

**Figure 7:**
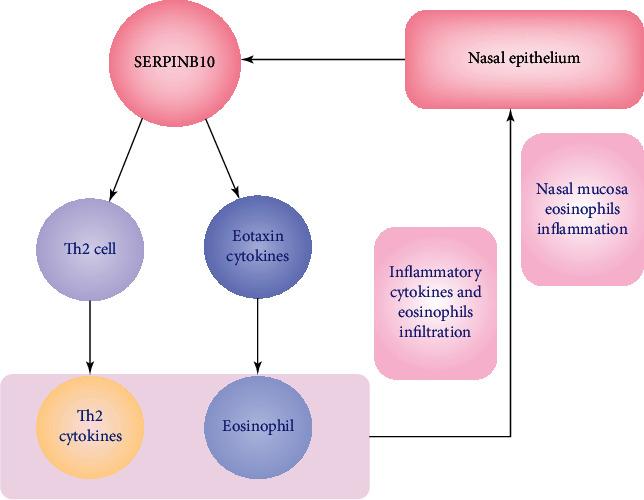
Epithelial-derived SERPINB10 could promote Th2 cell differentiation and induce tissue eosinophil recruitment, promote the infiltration of inflammatory cytokines and eosinophil infiltration into nasal mucosal tissue, and then exacerbate the tissue eosinophilic inflammation. Th2: T helper 2.

Accordingly, overaccumulations of tissue eosinophils and type 2 cytokines were the major pathological features of CRSwNP, and epithelial-derived cytokines might be crucial for Th2-type cell recruitment and eosinophil infiltration [25–28]. SERPINB10, a proinflammatory cytokine, was demonstrated to be upregulated in bronchial epithelial cells and could promote the Th2 inflammatory response in asthma patients and murine models [29]. Mo et al. [18] found that increased expression of epithelial-derived SERPINB10 could promote airway eosinophil inflammation by regulating the expression of eotaxin cytokines, which play an essential role in the migration of eosinophils and promote airway eosinophil inflammation [30, 31]. In addition, a recent study demonstrated that SERPINB10 could aggravate house dust mite-induced airway inflammation and the Th2 inflammatory response, suggesting that SERPINB10 might be a pivotal regulator in airway inflammation [17]. In this study, we observed that tissue SERPINB10 expression was obviously increased in the CRSwNP patients and positively correlated with peripheral and tissue eosinophil counts and percentages. Intriguingly, no statistical difference was observed in SERPINB10 expression between CRSsNP patients and HCs, suggesting that SERPINB10 might primarily promote Th2 but not Th1 inflammatory response, which contributed to the pathogenesis of CRSwNP [32–34]. Thus, combining previous studies with our results, we hypothesized that increased SERPINB10 in nasal mucosa could promote Th2 cells differentiation and induce tissue eosinophils infiltration and recruitment, which aggravated the inflammation of nasal mucosa in CRSwNP (Figure 7).

Although FESS is an effective treatment that rapidly alleviates clinical symptoms, a large proportion of CRSwNP patients require repeated surgery due to recurrence, particularly for those with a higher degree of tissue eosinophil infiltration [35–37]. Thereby, identifying biomarkers to early predict the recurrence of CRSwNP is extremely important and necessary. Prior studies reported that epithelial SERPINB10 could promote eosinophilic inflammation and Th2 immune response in airway allergic disorders by inhibiting the apoptosis of Th2 cells [17, 18, 38]. Moreover, mucosal Th2 inflammation and eosinophil infiltration were predominated in the histopathology of CRSwNP, and the severity of type 2 inflammation and eosinophilic inflammation in tissues affected the prognosis and recurrence of CRSwNP [39, 40]. Lou et al. [41] found that the degree of tissue eosinophil infiltration was linked to the recurrence of CRSwNP after surgery when their levels surpassed a certain range. In the present study, we observed that tissue SERPINB10 levels were enhanced in recurrent patients than in primary patients. Moreover, tissue eosinophil counts and percentages also were clearly raised in recurrent CRSwNP patients and positively correlated with the elevation of SERPINB10 levels. These results indicated that tissue SERPINB10 mRNA levels and tissue eosinophil percentages were risk factors for CRSwNP recurrence, and tissue SERPINB10 levels showed a strong predictive ability for postoperative recurrence. Therefore, we speculated that tissue with higher levels of SERPINB10 could augment Th2 inflammatory response and promote eosinophil recruitment in polyp tissue, which leads to a worse prognosis and a higher risk of recurrence in CRSwNP. Ultimately, we suggested that tissue SERPINB10 appeared to be a promising indicator to predict relapse after surgery in CRSwNP patients.

There are some limitations in the present study. First, the number of recruited participants is relatively limited, which makes the statistical results less reliable. Second, no international consensus has been achieved on the diagnostic criteria for CRSwNP recurrence, which limits the generation of conclusions. Further investigations with a larger sample size are needed to obtain more convictive conclusions and reveal the mechanism of SERPINB10 in CRSwNP.

In summary, our data suggested that SERPINB10 might be associated with the pathogenesis of CRSwNP, and increased tissue SERPINB10 levels were associated with eosinophilic inflammation. Tissue SERPINB10 could be used to early predicting postoperative recurrence in CRSwNP patients. Further investigations are necessary to determine the effect of SERPINB10 on the pathology of CRSwNP and its underlying mechanisms of recurrence.

## Figures and Tables

**Table 1:** Baseline characteristics of all patients.

Variables	HCs (*n* = 40)	CRSsNP (*n* = 40)	CRSwNP (*n* = 60)	*p*
Age (years)	36.7 ± 9.9	39.6 ± 11.0	38.9 ± 11.8	0.476
Gender (male/female)	20/20	23/17	38/22	0.416
BMI (kg/m^2^)	24.0 ± 3.9	24.8 ± 4.4	24.2 ± 4.1	0.704
Allergic rhinitis (yes/no)	0/40	7/33	16/44	0.002
Asthma (yes/no)	0/40	4/36	9/51	0.040
Smoking (yes/no)	17/23	19/21	33/27	0.456
Drinking (yes/no)	7/33	11/29	15/45	0.541
Peripheral eosinophil count (10^6^/L)	130 ± 100	190 ± 90	200 ± 100	0.003
Peripheral eosinophil percentage (%)	1.7 ± 1.1	1.9 ± 0.9	2.7 ± 1.1	<0.001
VAS score	—	3.0 ± 1.8	4.6 ± 2.8	0.002
TNSS score	—	10.1 ± 3.9	15.1 ± 3.6	<0.001

HC: healthy control; CRSsNP: chronic rhinosinusitis without nasal polyps; CRSwNP: chronic rhinosinusitis with nasal polyps; BMI: body mass index; VAS: visual analogue scale; TNSS: total nasal symptom scores.

**Table 2:** Characteristics of primary and recurrent CRSwNP patients.

Variables	pCRSwNP group (*n* = 36)	rCRSwNP group (*n* = 24)	*p*
Age (years)	37.7 ± 12.2	40.1 ± 11.4	0.446
Gender (male/female)	22/14	16/8	0.662
BMI (kg/m^2^)	24.1 ± 4.4	24.4 ± 3.6	0.783
Allergic rhinitis (yes/no)	5/31	11/13	0.006
Asthma (yes/no)	3/33	6/18	0.077
Smoking (yes/no)	18/18	15/9	0.340
Drinking (yes/no)	9/27	6/18	1.000
Peripheral eosinophil count (10^6^/L)	100 ± 100	200 ± 120	0.226
Peripheral eosinophil percentage (%)	2.3 ± 1.1	3.0 ± 1.0	0.029
Tissue eosinophil count (n/HPF)	22.2 ± 10.3	30.2 ± 8.7	0.003
Tissue eosinophil percentage (%)	18.5 ± 6.2	27.7 ± 5.8	<0.001
VAS score	5.0 ± 2.1	6.1 ± 2.4	0.061
TNSS score	6.5 ± 2.9	6.3 ± 3.3	0.826
Lund-Mackay score	13.8 ± 3.4	17.0 ± 3.0	<0.001

CRSwNP: chronic rhinosinusitis with nasal polyps; pCRSwNP: primary chronic rhinosinusitis with nasal polyps; rCRSwNP: recurrent chronic rhinosinusitis with nasal polyps; BMI: body mass index; VAS: visual analogue scale; TNSS: total nasal symptom scores; HPF: high power field.

**Table 3:** Correlation between tissue SERPINB10 level and parameters in CRSwNP patients.

Parameters	*r*	*p*
Gender	-0.071	0.404
Age	0.162	0.056
BMI	0.253	0.051
Peripheral eosinophil count (10^6^/L)	0.338	0.008
Peripheral eosinophil percentage (%)	0.153	0.241
Tissue eosinophil count (n/HPF)	0.451	<0.001
Tissue eosinophil percentage (%)	0.394	0.002
VAS score	0.456	<0.001
TNSS score	0.248	0.056
Lund-Mackay score	0.321	0.013

CRSwNP: chronic rhinosinusitis with nasal polyps; BMI: body mass index; VAS: visual analogue scale; TNSS: total nasal symptom scores.

**Table 4:** Binary regression analysis for predictors of recurrence in CRSwNP patients.

Variables	OR	95% CI	*p*
Peripheral eosinophil count (10^6^/L)	1.001	0.994-1.008	0.715
Peripheral eosinophil percentage (%)	1.970	0.866-4.485	0.106
Tissue eosinophil count (n/HPF)	1.087	0.999-1.183	0.052
Tissue eosinophil percentage (%)	1.176	1.031-1.343	0.016
Tissue SERPINB10 level	1.982	1.146-3.428	0.014

CRSwNP: chronic rhinosinusitis with nasal polyps; OR: odds rate; CI: confidence interval; HPF: high power field.

**Table 5:** ROC analysis results of variables for predicting CRSwNP recurrence.

Variables	AUC	*p*	95% CI	Sensitivity	Specificity	Cut-off value
Tissue eosinophil percentage	0.741	0.002	0.612-0.871	0.708	0.806	25.60
Tissue SERPINB10 level	0.773	<0.001	0.625-0.921	0.791	0.917	2.34
Tissue eosinophil percentage+tissue SERPINB10 level	0.917	<0.001	0.845-0.989	0.958	0.833	—

ROC: receiver operating characteristic; AUC: the area under the curve; CI: confidence interval.

## Data Availability

Data will be available upon reasonable request.
